# Soluble and insoluble lysates from the human A53T mutant α-synuclein transgenic mouse model induces α-synucleinopathy independent of injection site

**DOI:** 10.21203/rs.3.rs-3982325/v1

**Published:** 2024-03-05

**Authors:** Michael Lee, Justin Barnes, Scott Vermilyea, Joyce Meints, Hector Martinez

**Affiliations:** University of Minnesota; University of Minnesota; University of Minnesota; University of Minnesota; University of Minnesota

## Abstract

Pathological aggregation of a-synuclein (aS) is implicated in the pathogenesis of Parkinson’s disease (PD) and other a-synucleinopathies. The current view is that neuron-to-neuron spreading of aS pathology contributes to the progression of a-synucleinopathy. We used an A53T mutant human aS transgenic mouse model (*TgA53T*) to examine whether the site of pathogenic aS inoculation affects the pattern of neuropathology and whether soluble and insoluble fractions derived from crude pathogenic tissue lysates exhibit differential capacities to initiate aS pathology. To test whether the inoculation site impacts the ultimate spatial/temporal patterns of aS pathology, aS preformed fibrils (PFF), or brain homogenates from *TgA53T* mice with a-synucleinopathy, were injected into the cortex/striatum, brain stem, or skeletal muscle. In all cases, inoculation of pathogenic aS induced end-stage motor dysfunction within ~100 days post-inoculation (dpi). Significantly, irrespective of the inoculation sites, ultimate distribution of the aS pathology was like that seen in normally aged *TgA53T* mice at end-stage, indicating that the intrinsic neuronal vulnerability is a significant determinant in the induction of aS pathology, even when initiated by inoculation of pathogenic aS. Temporal analysis of brain stem injected *TgA53T* mice show that initial aS pathology was seen by 30 days post-inoculation and inflammatory changes occur at later stages. To determine if the aS species with differential solubility are differentially pathogenic, brain lysates from end-stage *TgA53Tmice* were fractionated into highly soluble (S150) and insoluble (P150) fractions, as well as the endoplasmic reticulum (ER)-enriched fraction (P100). Significantly, all fractions were able to seed *de novo* aS pathology *in vivo*, when injected unilaterally into *TgA53Tmice* with the ER fractions being most pathogenic. Our results suggest that multiple aS species from brain can initiate the development of progressive aS pathology.

## Introduction

Parkinson’s disease (PD) is a common late-onset, progressive neurodegenerative disorder characterized by progressive degeneration of multiple neuronal populations, particularly the dopaminergic (DAergic) neurons of the substantia nigra pars compacta (SNpc), and the presence of intracellular aggregates of α-synuclein (αS) termed Lewy bodies (LBs) and neurites (LNs). While the etiology of PD is unknown in most cases, degenerating neuronal populations in PD exhibit α-synuclein (αS) abnormalities and mutations in the *αS* gene cause familial PD, indicating that the αS abnormalities are mechanistically linked to pathogenesis of PD and other α-synucleinopathies^1,2^.

The temporal analysis of αS pathology in human cases^3^ indicates that αS pathology in the PD brain originates in several focal sites, particularly the dorsal motor nucleus of vagus (DMV) and the anterior olfactory nucleus. With the preclinical progression of the disease, αS pathology moves anteriorly in a topographically defined manner until reaching the cortex during the symptomatic stage. This staging led to the hypothesis that αS pathology moves via cell-to-cell transmission of amyloidogenic αS species that serves as a conformational template to seed pathology. This hypothesis was also further supported by the presence of LBs in fetal dopamine neurons grafted into PD patients ^4,5^. Cell-to-cell transmission of α-synucleinopathy has received formal experimental support from brain injection studies establishing αS spreads transynaptically in mice^6^. Currently, direct inoculation of αS pre-formed fibrils (PFFs) or tissue lysates/fractions containing αS aggregates can induce αS pathology when directly injected into the central nervous system (CNS) or peripherally^7^. However, most current studies involve the use of αS PFF derived from recombinant αS. Thus, it is unknown if the spatial-temporal pattern of αS pathology are comparable following inoculation of various pathogenic αS species to various brain regions of αS Tg models.

To better understand the spreading of α-synucleinopathy, we directly compared spatial-temporal pattern of αS pathology following inoculation of pathogenic αS species into different brain regions (cortex/striatum and dorsal brain stem) and skeletal muscle of A53T mutant human αS (*TgA53T*) transgenic mice^8^. We show that all three inoculation sites lead to end-stage pathology that is indistinguishable to that observed with aging *TgA53T* mice, indicating that specific neuronal populations are predisposed to the effects of toxic αS species *in vivo*. Temporal analysis show that overt neuroinflammation occurs after onset of αS pathology, supporting the view that inflammation is not the primary driver for the initial onset and spreading of αS pathology. We also show that both the soluble and insoluble fractions from end-stage *TgA53T* mice can initiate pathology in younger *TgA53T* mice. Further, endoplasmic reticulum (ER) associated αS from the end-stage *TgA53T* mice induces pathology sooner than other lysates or αS PFF. Thus, multiple species of αS are capable of inducing pathology *in vivo* and suggest the need to separate such entities to further clarify the form of αS that is necessary to seed pathological alterations *in vivo*.

## Results

### Lysates from symptomatic TgA53T mice and αS PFF induce α-synucleinopathy with similar end-stage distribution.

**Figure 1:**
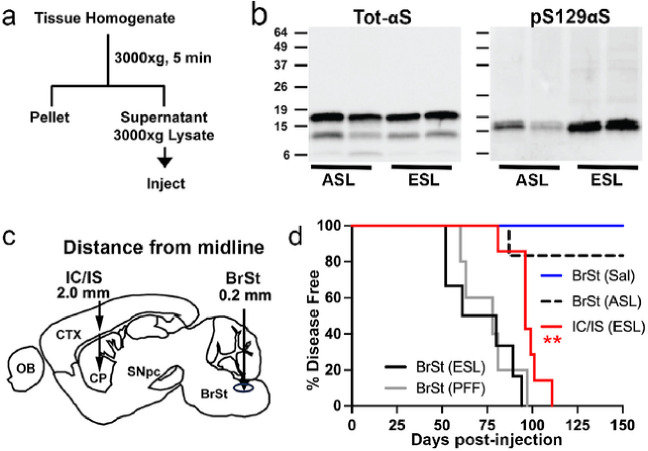
Injection of *TgA53T* brain-derived lysate into cortex/striatum or brain stem induces α-synucleinopathy and premature death in young *TgA53T* mice. ***a)*** Schematic representation of the protocol used to generate the injected ESL and asymptomatic ASL. ***b)*** Analysis of ASL and ESL for total αS (top) and pS129 αS levels. While similar levels of total aS is present in ASL and ESL, ESL contains more pS129 aS than ASL. ***c)*** Diagrams depicting the stereotaxic injection sites for the IC/IS and BrSt injection models. Stereotaxic coordinates: IC/IS 2.0 mm lateral from the midline, +0.2 mm relative to bregma, and 0.8 and 2.6 mm deep from the dura; BrSt: 0.2 mm lateral from the midline, −7.34 mm from bregma, 3.75 mm deep from the dura. ***d)*** Kaplan-Meier survival curve of mice IC/IS-injected with ESL and BrSt-injected animals with aS PFF, ESL, ASL, and saline. Control animals receiving saline and ASL all survived disease-free to 200 days post injection (dpi), excepting one animal injected with ASL that developed disease and died 136 dpi. **p<0.01, IC/IS ESL vs BrSt ESL or BrSt PFF, Mantel-cox log-rank test. All n=6 except BrSt (PFF), n=5. Abbreviations: alpha synuclein, aS; asymptomatic lysate, ASL; end-stage lysate, ESL; intracortical/intrastriatal, IC/IS; brainstem, BrSt; preformed fibril, PFF.

Although the intracortical/intrastriatal (IC/IS) transmission model shows the induction and spread of αS pathology in *TgA53T* mice^6^, studies show that injections of pathogenic αS lysates or preformed αS fibrils (PFF) in multiple areas, including the skeletal muscle, can induce widespread αS pathology over time^9^. Thus, we examined whether the inoculation site impacts the onset and spread of αS pathology in *TgA53T* mice. Because brain stem (BrSt) neurons are affected early by αS pathology in both human PD^10^ and in the *TgA53T* mouse model^8^, we compared the disease produced by the IC/IS inoculation with the BrSt injections of αS PFF. We injected young, disease-free 3–6 month-old *TgA53T* mice (all from line G2–3 unless noted) with end-stage lysate (ESL or S3000, a crude 3000×g pooled lysate) from BrSt/Spinal Cord (SpC) of end-stage (ES) affected *TgA53T* mice or lysates from disease-free (asymptomatic lysate, ASL) 5–6 months old *TgA53T* mice (Fig. 1a–c). Immunoblot analysis (Fig. 1b) show that both ASL and ESL contain similar amount of total αS but ESL contains greater amount of pS129αS, consistent with pathology in ES *TgA53T* mice. Like that seen by Luk *et al*. (2012), the mean of survival for bilateral IC/IS injected mice was 98 ± 8.6 days post-inoculation (dpi) (Mean ± SD) (Fig. 1d, Supplemental Fig. 1). Unilateral inoculation of ESL into the BrSt resulted in earlier onset of motor dysfunction with the mean survival of 71.3 ± 18.7 dpi (Fig. 1d, S1*a*), a significantly shorter disease time course than with IC/IS dual injections. Inoculation of αS PFF into BrSt leads to mean survival time (77.4 ± 13.16 dpi, n = 5) that is like the ESL inoculation (Fig. 1d, S1*a*). All the mice injected with ASL or saline remained disease free at ~ 150 dpi (Fig. 1d) except one ASL-inoculated animal who succumbed to disease prematurely died at ~ 9 months of age, a time point where normal aged *TgA53T* mice start to show disease^8^. Thus, it is likely that this animal developed disease independently of the injected lysate.

**Figure 2:**
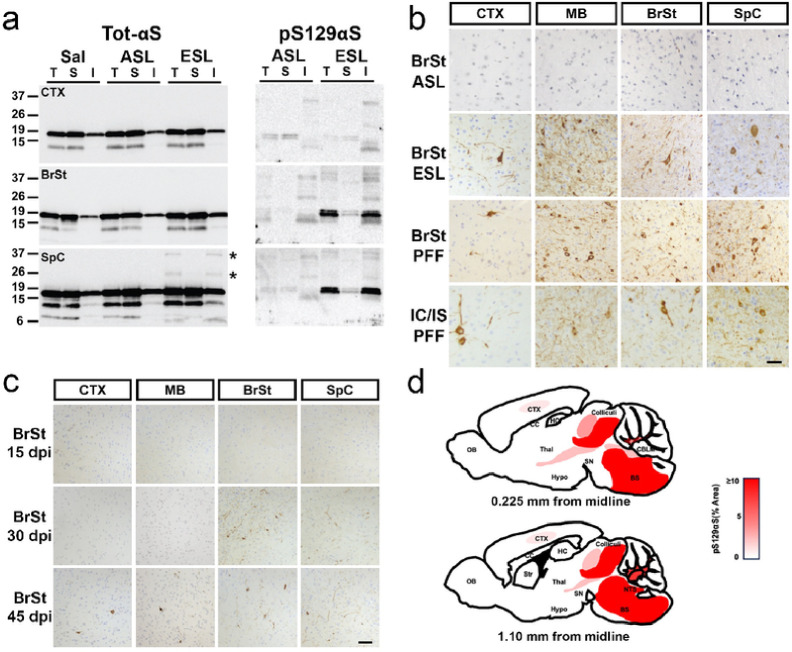
Aggregation of αS is induced by ESL in *TgA53T*mice with pathological distribution that is independent of injections site. ***a)*** Accumulation of pathological HMW aS species (*) and pS129 αS in affected brain regions in detergent insoluble faction. Total lysates (T) from unaffected CTX and affected BrSt and SpC from saline, ASL, and ESL of BrSt-injected animals were separated into TX-soluble (S) and TX-insoluble (I) fractions. Detection of total αS (left) demonstrates the presence of HMW insoluble αS species in SpC (*) ESL-inoculated mice. Immunoblots for pS129 αS clearly show a complete lack of pathological αS in ASL-injected animals and CTX of ESL-inoculated mice. Virtually all of HMW aS species (*) and pS129 aS partitions with the TX-insoluble fraction in BrSt and SpC. ***b)*** Aggregated aS, marked by pS129 aS immunoreactivity, is abundant in animals injected with ESL or aS PFF. ***c)*** Immunoreactivity for pS129 αS occurs between 15- and 30-days post BrSt injection of ESL. ***D)*** Diagrams depicting the distribution of αS pathology as observed by immunohistochemical analysis. Shades of red denote the relative abundance of αS inclusions (% Area covered by pS129 aS). Note that the sections are unilateral to the injection site even though pathology is detected bilaterally. Abbreviations: alpha-synuclein, aS; end-stage lysate, ESL; high molecular weight, HMW; cortex, CTX; brainstem, BrSt; spinal cord, SpC; asymptomatic lysate, ASL; triton-X, TX; preformed fibril, PFF. Scale bars: 50 μm.

Biochemical analysis of the ESL inoculated animals confirms that the ESL, but not ASL, leads to αS pathology (Fig. 2a). Analysis of Triton X100-detergent soluble and insoluble fractions reveal the accumulation of insoluble high molecular weight (HMW) species of αS (Fig. 2a, asterisks) in the SpC of ESL injected mice. In the ESL injected mice, insoluble pS129αS accumulates in BrSt and SpC, corresponding to the regions most affected by αS pathology (Fig. 2a). Consistent with lack of αS pathology, pS129αS does not accumulate in ASL (Fig. 2a) or Saline (not shown) injected mice. This pattern of αS pathology is equivalent to that seen with the ES *TgA53T* mice that normally develop α-synucleinopathy from aging^8^.

Immunohistochemical detection of pS129αS demonstrates robust staining in multiple brain tissues from IC/IS- and BrSt-injected end-stage animals (Fig. 2b). Consistent with the lack of motor phenotype, no pathological alterations are noted in ASL-inoculated mice (Fig. 2b) or saline-injected animals (data not shown). Significantly, despite the differences in the site of inoculation (IC/IS vs BrSt), the overall patterns of pathology in the ES mice were comparable to each other with most abundant pathology in the BrSt and SpC (Fig. 2b), a pattern very similar to what is seen in the *TgA53T* mice that naturally develop αS pathology with aging^8^. However, we noted that IC/IS injected animals exhibit more pS129αS pathology in CTX compared to BrSt injected animals (Fig. S1*b*). To determine if components of lysates other than pathogenic αS could be responsible for the distribution of the pathology, we also examined animals that were BrSt injected with αS PFF. Our results show that the pattern of pathology seen in the ESL inoculated animals is very similar to animals injected with αS PFF into BrSt (Fig. 2b, S1*b*). Moreover, the pattern of αS pathology induced by BrSt injections are also very similar to the pathology achieved in this line of mice by peripheral, intramuscular (IM) inoculation of αS PFF^9,11^. To determine if the prominent subcortical pathology is unique to *TgA53T(G2–3)* line or occurs in another line of *TgA53T*, we performed IC/IS injections on *TgA53T(H5),* which express 50% less αS than *TgA53T(G2–3)*^8,12^. Because of lower transgene expression, injected *TgA53T(H5)* animals developed progressive motor phenotype later (~ 150 dpi) than the *TgA53T(G2–3)* (~ 100 dpi) following IC/IS injections of αS PFF. Neuropathological analysis shows that, as with *TgA53T(G2–3),* αS pathology at ES mice was most abundant in BrSt/SpC region (Fig. S2). Significantly, the *TgA53T(H5)* animals also showed a more widespread distribution of αS pathology, including αS pathology in the hippocampus (Fig. S2*a*). In summary, in all animals inoculated with ESL or αS PFF showed most severe BrSt and SpC αS pathology, regardless of initial sites of injections.

Collectively, these results indicate that in *TgA53T* mice, the ES distribution of αS pathology is independent of inoculation site and likely determined by factors other than local seeding by pathogenic αS. Moreover, because our Tg models show highest levels of transgene expression in the CTX^8,11,13^, relative lack of forebrain pathology is not because of insufficient transgene expression in this area. While previous studies with the IC/IS injection model show that cortical and striatal αS pathology occurs prior to BrSt or SpC^6^, we observe more prominent early subcortical pathology with the BrSt inoculation. Thus, following the BrSt inoculation, the appearance of pS129αS over time shows that initial αS pathology develops proximal to the inoculation site where αS pathology is seen in the pons by 30 dpi (Fig. 2c). By 45 dpi, regions rostral and caudal to the BrSt inoculation sites exhibits αS pathology (Fig. 2c). Thus, we propose that following BrSt injection of pathogenic αS, the pathology spreads both rostral and caudal directions but most prominent pathology occurs in subcortical regions (Fig. 2d). Analysis of IC/IS injected *TgA53T(H5)* animals at intermediate stage (90 dpi) shows the presence of pS129αS in the CTX but more obvious pS129αS pathology is seen in the BrSt and SpC areas (Fig. S2b). Overall, current results collectively show that regardless of the initial injection site, subcortical areas (e.g. BrSt, SpC) exhibit earliest αS pathology and culminates in most severe αS pathology at ES *TgA53T* mice.

### Neuroinflammation follows onset of α-synucleinopathy.

**Figure 3:**
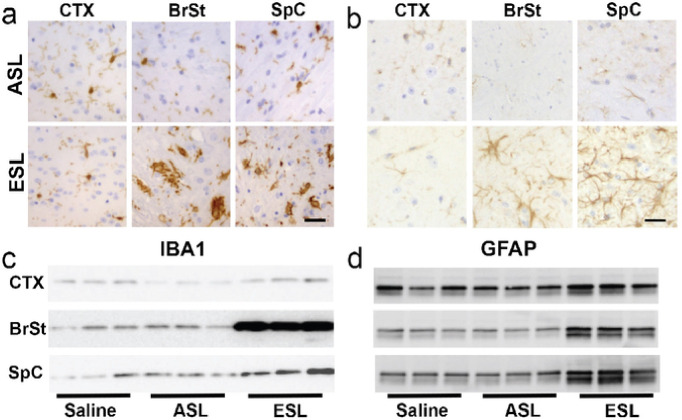
Neuroinflammation is increased in affected brain regions. ***a,b)*** Tissue sections stained for Iba1 ***(a)***and GFAP ***(b)***. BrSt and SpC from ESL-injected *TgA53T* mouse reveal abundant activated microglia and astrocytes. Areas lacking αS pathology, such as the CTX, as well as animals injected with ASL, are free of glial activation. ***c,d)*** Biochemical analysis of whole-tissue lysates from CTX, BrSt, and SpC of saline-, ASL-, and ESL-injected mice show robust increases in Iba1 ***(c)*** and GFAP ***(d)***in BrSt and SpC or ESL injected mice. Quantitation of the blots are shown in Fig. S3. Abbreviations: alpha synuclein, aS; brainstem, BrSt; spinal cord, SpC; end-stage lysate, ESL; cortex, CTX; asymptomatic lysate, ASL. Scale bars: 25 μm.

It has been proposed that inoculation with pathogenic αS could lead to early neuroinflammation that may promote further propagation of α-synucleinopathy^14^. Thus, we examined the spatial-temporal relationship between αS pathology and neuroinflammation. First, we confirmed that at ES, animals following BrSt injection exhibit coincident αS pathology and neuroinflammation. Tissue sections from BrSt-injected animals were stained for microglia (Iba1; Fig. 3a) and astrocytes (GFAP; Fig. 3b). Based on the morphology of the cells, no obvious neuroinflammatory changes are seen in absence of significant αS pathology, such as in CTX (Fig. 3a,b) and in animals injected with saline (not show) or ASL (Fig. 3a,b). However, in ES animals following ESL injection, areas with significant αS pathology (BrSt, SpC) exhibit abundant microglia and astrocytes with highly activated morphology. Immunoblotting for Iba1 and GFAP reveals no change in Ctx, but a significant increase in BrSt and SpC of ESL-injected mice at ES disease (Fig. 3c,d; Fig. S3).

**Figure 4:**
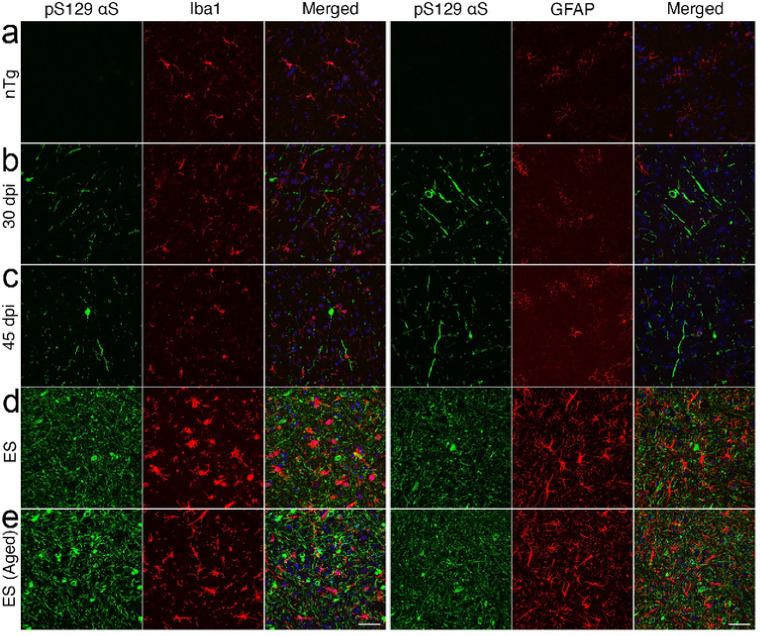
Neuroinflammation occurs after αS pathology in *TgA53T* mice. BrSt sections from Control mice ***(a, ntg)*** and *TgA53T* mice injected with ESL into BrSt were analyzed at 30 dpi ***(b)*** 45 dpi ***(c)***, and ES **(*d*, injected; *e*, aged**). The sections were stained for pS129 aS and Iba1 or GFAP. Aggregated aS (pS129 aS) is clearly seen in 30- and 45-dpi samples ***(b, c)*** but the overall morphology and density of Iba-1 and GFAP staining is comparable to nTg mice ***(a)***, indicating that the presence of pS129 aS is not accompanied by obvious glial activation. In contrast, there is a clear increase in Iba1 and GFAP staining with well-established pS129 aS pathology in ES animals ***(d, e)***. Abbreviations: alpha synuclein, aS; brainstem, BrSt; end-stage lysate, ESL; end-stage, ES; non transgenic, nTg. Scare bar: 50 mm

To better determine the spatial-temporal relationship between the onset of microglial activation and αS pathology, the sections from BrSt inoculated mice harvested at 15-, 30-, and 45-dpi that were evaluated for αS pathology (Fig, 2c) were stained for microglia (Iba-1) and astrocytes (GFAP) (Fig. S4). With BrSt inoculation, αS pathology is seen in the pons by 30 dpi (Fig. 2c). However, there is no obvious signs of astrocytic or microglial activation at 30- or 45-dpi (Fig. S4). Because αS pathology is still sparse at 45 dpi, we performed double immunofluorescence analysis of pS129αS with Iba1 or GFAP (Fig. 4) to determine if presence of αS pathology could be associated with local changes in inflammatory changes. Double immunofluorescence analyses show that that while αS pathology in ES mice are clearly associated with nearby reactive astrocytes and microglia, such glial responses do not accompany αS pathology at 30- or 45-dpi (Fig. 4b,c). Similar analysis of early-stage mice following IM αS PFF injections show that initial onset of pS129αS pathology in SpC, occurring as early as 15–30 dpi, is not accompanied by obvious glial activation (Fig. S5). While other A53T Tg models show αS pathology in astrocytes^15,16^, we do not observe significant pS129αS in astrocytes at ES mice (Fig. 4)^17^.

Collectively, our results indicate that glial activation, particularly activation of microglia, is only evident after substantial αS pathology is established. Similar pattern of initial αS pathology followed by neuroinflammation was reported with IM inoculation of M83 line^18^. Thus, while microglia and astrocytes may modulate neuronal spreading of αS pathology or neuronal survival, our results indicate that it is unlikely that the neuroinflammation is a significant factor in the initial onset of αS pathology in *TgA53T* model of α-synucleinopathy.

### Both highly soluble and insoluble fractions induce αS pathology

**Figure 5:**
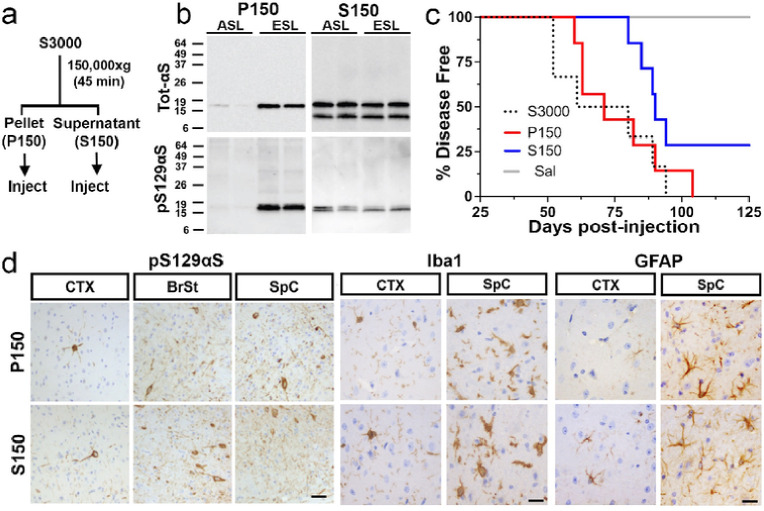
Both soluble and insoluble fractions from ESL induces a-synucleinopathy in TgA53T mice. ***a)*** Flow diagram showing the separation of S3000 into soluble (S150) and insoluble (P150) fractions. ***b)*** Biochemical analysis of S150 and P150 fractions derived from ASL and ESL. Equal amount of total protein was analyzed. P150 fractions from ASL contain very little aS while total aS and pS129 aS are abundant in P150 fractions from ESL. Despite the abundant total aS in S150, much lower pS129 aS is present in the S150. ***c)*** Kaplan-Meier survival curve of mice injected with S150 and P150 into BrSt. Also shown are the curve for the S3000-ESL injected mice shown in Fig. 1d. Both S150 and P150 injections lead to motor dysfunction and reduced life span. S3000, n=6; P150, n=7; S150, n=7; Sal, n=6. ***d)***Neuropathological analysis of affected S150 and P150 injected animals show expected distribution of aS pathology (pS129 aS) and corresponding neuroinflammation (Iba1 and GFAP). Abbreviations: end-stage lysate, ESL; asymptomatic lysate, ASL; alpha synuclein, aS. Scale bars: 50 μm for pS129 aS, 25 μm for Iba1 and GFAP.

Because αS fibrils and disease associated aggregates in *TgA53T* mice are detergent insoluble^9,13^, we tested whether the induction of αS pathology by the ESL is mediated by the insoluble αS species. The pathogenic S3000 ESL was centrifuged at 150,000×*g* to obtain highly soluble (supernatant, S150) and insoluble (pellet, P150) fractions (Fig. 5a). Biochemical analyses of the fractions show that very little αS is found in P150 from asymptomatic animals (Fig. 5b). Moreover, most of the pS129αS, representing the overt αS pathology, is highly enriched in the P150 fractions from the symptomatic mice (Fig. 5b). In contrast, the amount of total αS is the same in the S150 fractions, regardless of the disease state of the animal (Fig. 5b). Despite the high levels of total αS, the levels of pS129αS in S150 is lower than in the P150 with no differences between the disease state of the animal (Fig. 5b). Thus, we expected that αS pathology would be selectively induced by the P150-ESL fraction.

Following BrSt injections of P150 and S150 fractions, we were surprised to find that, regardless of the solubility of the inoculated material, inoculated *TgA53T* mice developed motor dysfunction leading to premature death with average lifespans of 75 ± 7 dpi for P150 fraction and 88 ± 3 dpi for S150 fraction (Fig. 5c). While 2 out of 7 subjects injected with S150 did not develop disease phenotype by 150 dpi, for those animals that died prematurely, there was no differences in the average lifespan (Fig. 5c, *p* = 0.1397) between S150 and P150 injected groups.

Histological (Fig. 5d) and biochemical (Fig. S6) analyses of ES P150- and S150-injected animals demonstrate virtually indistinguishable αS pathology both in the spatial pattern and severity of αS aggregation (compare Figs. 2 and 5, S6). Aggregation of αS occurred throughout the CNS but was most robust in the BrSt and SpC (Fig. 5d). Biochemical analysis of brain tissue also reveals accumulation detergent insoluble αS species in the insoluble fraction from BrSt and SpC (Fig. S6a) and dramatic increases in pS129αS in both BrSt and SpC (Fig. S6*b*). Finally, based on immunostaining for Iba1 and GFAP, the distribution and intensity of neuroinflammation is similar in S150- and P150-BrSt-injected animals (Fig. 5d), and is nearly identical to that observed in ESL-injected mice (see Fig. 3). Thus, by three broad metrics: survival, IHC, and biochemistry, the α-synucleinopathy and disease induced by injection of all three end-stage lysates (S3000, S150, P150) are essentially identical.

Since S3000 ASL does not induce disease and there is no obvious differences between S150 from ASL and ESL on our immunoblot analysis, S150 from ESL may contain soluble pathogenic αS conformers that is SDS labile. Thus, incomplete penetrance of end-stage S150 to cause disease could be due to the possibility that toxic αS assemblies in S150 fractions are more prone to degradation following cellular uptake compared to more mature insoluble toxic αS species in P150 (Fig. 5b). As an initial test of this hypothesis, we performed dot blot analysis of P150 and S150 for αS oligomers, using FILA1^19,20^ and OC antibody^21^ (Fig. S7*a,b*). We previously showed that FILA1 can recognize both soluble and insoluble αS aggregates derived of *TgA53T* model^20^ and OC antibody selectively recognizes mature fibrils^21^. Our results show that while levels of FILA1 + oligomers are similar between P150 and S150, the levels of OC + oligomers are significantly more abundant in P150 (Fig. S7*a,b*). We also subjected S150 and P150 to proteinase K treatment as more compact mature aggregates should be more resistant to proteinase K treatment. The results show that αS in P150 is more resistant to proteinase K proteolysis than αS in S150 (Fig. S7*c*). Thus, we conclude that the presence of FILA1 + oligomers/aggregates in both S150 and P150 are pathogenic. Further, more labile FILA1 + oligomers in S150 may be responsible for the partial penetrance of S150 in inducing disease.

### Microsomes from symptomatic TgA53T mice induce rapid α-synucleinopathy.

We previously showed that α-synucleinopathy in *TgA53T* mice is associated with accumulation of αS oligomers/aggregates in endoplasmic reticulum (ER) and chronic ER stress contributes to neurodegeneration^13,20^. Analyses of *TgA53T* mice inoculated with S3000 ESL show that αS pathology in these mice are also associated with signs of chronic ER stress (Fig. S8), suggesting that ER stress occurs even when α-synucleinopathy is induced by exogenous inoculation of pathogenic αS. We recently showed that ER enriched microsomes from *TgA53T* mice, containing αS aggregates, are highly toxic to cultured neurons and aggressively induces αS aggregates and cell death in cultured neurons^22^. Thus, we examined whether the microsome fraction from symptomatic *TgA53T* mice can induce pathology following BrSt injection.

**Figure 6:**
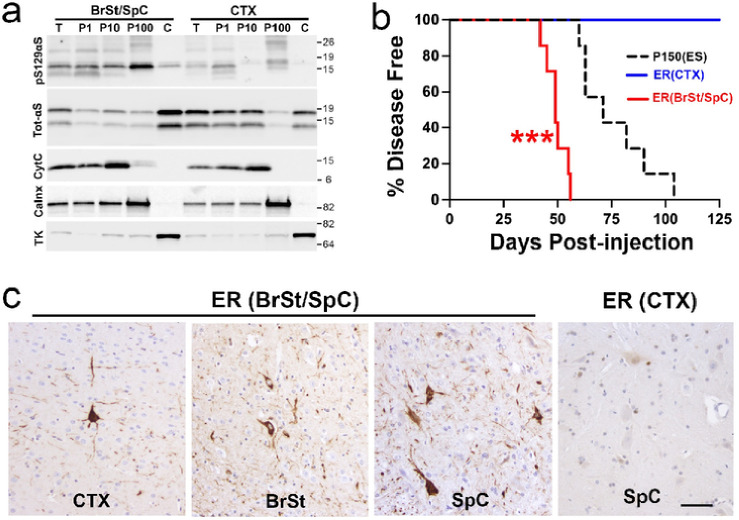
ER-enriched fraction from ES *TgA53T* mice, containing ER-associated aS, is highly pathogenic. ***a)*** Subcellular fractions from BrSt/SpC and CTX were analyzed for pS129 aS, total aS, and organelle markers (Cytochrome C, CytC-mitochondria; Calnexin, Calnx-ER; Transketolase, KT-Cytosol). Calnexin-enriched ER fraction (P100) is relatively free of CytC and TK. pS129 aS is enriched in P100 from BrSt/SpC. ***b)*** Kaplan-Meier survival curve of mice injected with P100(ER) fractions from BrSt/SpC (BS/SC) or CTX. For reference, curve for P150 injected animals from Fig. 4c is also shown. ***p<0.001, ER(BrSt) vs P150(ES), Mantel-cox log-rank test. P150(ES), n=7; ER(BrSt/SpC), n=7; ER(CTX), n=6. ***c)***Neuropathological analysis show that ER(BS/SC) fraction induces pS129 aS pathology in *TgA53T* mice, particularly in BrSt and SpC. In contrast, ER(CTX) fraction do not cause pS129 pathology. Abbreviations: endoplasmic reticulum, ER; end-stage, ES; alpha synuclein, aS; brainstem, BrSt/BS; spinal cord, SpC/SC; cortex, CTX. Scale bar: 50 μm.

Microsomes from pooled BrSt/SpC or CTX from the symptomatic *TgA53T* mice were used for BrSt injections of 3–4 mos old *TgA53T* mice. Analysis of the fractions show that pS129αS is enriched in microsomes (P100 or ER) from BrSt/SpC where very little pS129αS is seen in P100 from CTX (Fig. 6a). Analysis of organelle markers show that P100 fraction is also enriched in the ER marker (Grp78/BiP). Analysis of injected animals show that P100 from symptomatic *TgA53T* mice develop progressive motor abnormalities by ~ 50 dpi while the animals inoculated with P100 from CTX did not show any disease phenotype (Fig. 6b). Significantly, microsome-inoculation leads to onset of motor symptoms much faster than any of the other fractions tested in this study (Compare Figs. 1d,5c & 6b), indicating that microsome associated αS oligomer/aggregates are highly pathogenic *in vivo*. Neuropathological analysis show that the early motor deficits were indeed associated with significant αS pathology (Fig. 6c) with the pattern that is seen with other αS fractions. Collectively, these results provide *in vivo* confirmation of highly pathogenic nature of ER-associated αS oligomer/aggregates^20,22^.

## Discussion

In this report, we provide a comparative analysis of αS pathology induced by inoculation of lysates from *TgA53T* mice. Specifically, we examined the induction of αS pathology as a function of different lysate solubility and location of inoculation. Like that reported with αS PFF models^15^, we show that regardless of inoculation site, pathogenic lysates from brains containing α-synucleinopathy lead to a remarkably consistent pattern of ES pathology in the *TgA53T* model. This indicates that the regional differences in the vulnerability to develop αS pathology with aging is maintained even when α-synucleinopathy is initiated by inoculation of pathogenic αS species. We also show that, *in vivo*, there are multiple αS species with different solubility that induces very similar αS pathologies.

Following the creation of the staging hypothesis of PD pathology by Braak and colleagues^3^, series of *in vitro* and *in vivo* studies have confirmed that αS pathology can spread from an initial site of αS aggregate induction to other neurons and brain regions^7,23^. It is also established that lysates from murine or human tissues with αS pathology can induce early αS pathology in αS Tg mouse models^6^. Further, numerous studies have shown that injections of αS PFF into CNS or peripheral tissue can induce αS pathology in both αS Tg mice and wild type mice^7^. While studies have generally assumed that the spreading of α-synucleinopathy occurs along the interconnected networks^23^, it is notable that while human α-synucleinopathy may progress rostrally from DMV, the interconnected aspect has not been demonstrated. Further, some studies in rodents have noted deviations from the simple cell-to-cell transmission model^15^. In this regard, we also show that while progressive α-synucleinopathy is induced by inoculation with pathogenic αS species, the pattern of α-synucleinopathy following different sites of injections is remarkably consistent with BrSt and SpC neurons being more vulnerable to developing α-synucleinopathy. Our time course study also shows that at intermediate stages, independent of where pathogenic αS is injected, αS pathology is more prominent in BrSt and SpC neurons.

Analysis of intermediate stage mice for coincidence of αS pathology and glial activation indicates the αS pathology occurs prior to the microglial or astrocyte activation. This result agrees with a prior study demonstrating a temporal relationship between onset of αS pathology and microglial activation following IM inoculation^18^ and we have extended prior studies by showing that at even at early stages of αS pathology, there is no obvious glial activation near the αS pathology (Fig. 4, S5). Thus, while microglia may promote αS propogation^14,24^, inflammatory activation of glial cells that can also influence the propagation of αS pathology likely to occur at more advanced stages of αS pathology.

Most *in vivo* studies on transmission of α-synucleinopathies have relied on αS PFFs or oligomers derived from recombinant αS as the initiating factor for pathology. Although PFF can efficiently induce pathology, the biological relevance of an *in vitro* aggregated recombinant αS is unclear as the structure of αS PFF could be divergent from *in vivo* pathology. Because toxic species of αS in human PD and *in vivo* models, such as the TgA53T model, are likely to be a complex mixture of various αS species^25–27^, characterizing the pathology induced by tissue-derived αS species will have relevance for *in vivo* pathogenesis of α-synucleinopathy. This view is supported by differential induction of pathology in huαS Tg models by various human brain derived lysates^28^. In *TgA53T* models, αS forms soluble oligomers, particularly in the ER, and increasingly aggregate to become insoluble as the disease progresses^13,20^. This, taken together with the fact that pathogenic αS PFF are insoluble, we initially expected that the insoluble fraction from ESL will contain most of the disease initiating activity. Thus, we expected P150, enriched in mature pS129αS containing aggregates, to aggressively induced αS pathology following inoculation. Surprisingly, the S150 from ES mice, despite being biochemically similar to the S150 from AS mice, is capable of inducing αS pathology *in vivo*. Although the exact nature of the soluble “toxic” species is unknown, our results indicate that S150 contains potentially pathogenic FILA1 + αS oligomers. Regardless, our results show that different species of αS with varying degrees of solubility can induce identical pattern of pathology. We also show that, consistent with the pathogenic importance of ER associated αS pathology^20,22^, microsomes from ES *TgA53T* mice are highly pathogenic in vivo. These data thus indicate that further separating soluble and insoluble αS species by size and/or subcellular localization could be a fruitful approach for identifying the elusive transmissible αS species.

In summary, our study indicates that differential neuronal vulnerability contributes to patterns of α-synucleinopathy. Thus, simple spreading of αS pathology via cell-to-cell connection cannot fully account for the progression of α-synucleinopathy. Further, our evidence supports the view that multiple αS species are capable of initiating αS pathology *in vivo* and it will be important to fully define the disease relevance of these different pathogenic αS species.

## Materials and Methods

### Transgenic mouse lines

The transgenic mouse line over-expressing A53T mutant human αS under control of the mouse prion protein promoter has been described previously (*TgA53T*, line G2–3 and line H5)^8^. *TgA53T(G2–3)* mice used develop a progressive, fatal neurological disease at ~ 10–14 months of age and show marked αS pathology by end-stage disease. *TgA53T(H5)* line express less αS (~ 50%) and do not spontaneously develop pathology^8^. Young, asymptomatic mice aged 3–6 months were used for all experiments. Animals showing complete hind-limb paralysis are considered end-stage. All study methods were approved in full by the University of Minnesota Institutional Animal Care and Use Committee and were consistent with the National Institutes of Health Office of Laboratory Animal Welfare Policy.

### Inoculation material

Lysates used for all injections were generated from tissues of *TgA53T* mice at either 4 months of age (asymptomatic lysate, ASL) or end-stage (end-stage lysate, ESL). BrSt and spinal cord (SpC) were combined and processed together, as both regions demonstrate robust αS pathology at end-stage^8^. Preparation of the 3000×g lysate (S3000) has been detailed previously^6^. Briefly, affected tissue previously harvested and stored at −80°C was sonicated in sterile saline (0.9% NaCl; 1:10 w/vol). The homogenate was centrifuged for five minutes (3000×g, 4°C) and the recovered supernatant was the 3000×g lysate (S3000). To obtain highly soluble (S150) and insoluble (P150) fractions, S3000 fraction was centrifuged for 45 minutes (150,000×g, 4°C). The resulting supernatant was termed the S150 fraction. The pellet was washed and resuspended in sterile saline by sonication (3×10s pulses) in half of the original volume ultracentrifuged and used as the P150 fraction.

Endoplasmic Reticulum (ER)-enriched fractions were obtained from *TgA53T* mice as previously detailed^20,22^. Briefly, fresh tissues were homogenized in a 1:10 (wt/vol) volume of lysis buffer (250 mM sucrose, 20mM HEPES, 10mM KCl, 1.5mM MgCl_2_, 2 mM EDTA, protease-inhibitor cocktail) and centrifuged at 1,000×g. The supernatant was centrifuged at 10,000xg to pellet mitochondria and the supernatant was centrifuged at 100,000xg to obtain ER (pellet) and cytosol (supernatant).

Human αS preformed fibrils (PFF) was generated from in vitro aggregation of recombinant wild type human αS monomer as previously described^9^. Human αS PFF were stored at 5 mg/ml and were sonicated and diluted to desired concentration prior to use.

### Inoculation of TgA53T mice

All injections were performed unilaterally into the right hemisphere in *TgA53T* mice at 3–6 months of age. Animals were stereotaxically injected with 2.5 μg of total protein in 2.5 μl. The injections occurred at a rate of 0.1 μl per minute using a 28g needle attached via tubing to a Hamilton syringe controlled by a constant pressure syringe pump (Harvard Apparatus, Holliston, MA). The needle was kept in place for five minutes post-injection. The stereotaxic coordinates used for the injection sites were as follows: intracortical/intrastriatal (IC/IS): 2.0 mm lateral from the midline, + 0.2 mm relative to bregma, and 0.8 and 2.6 mm deep from the dura; BrSt: 0.2 mm lateral from the midline, −7.34 mm from bregma, 3.75 mm deep from the dura. Intramuscular (IM) injections of αS PFF was done as described ^9^. Briefly, isoflurane anesthetized mice (~ 3.5 months old) were bilaterally injected into the *bicep femoralis* muscle bilaterally with 5 μg of PFF.

Starting at 60 days following PFF injections, the mice were evaluated for disease onset and progression three days per week (Monday, Wednesday, Friday). Disease onset was identified by an imbalance in gait leading to a wobbling phenotype and the end-stage was characterized as complete hindlimb paralysis. Animals were euthanized at either pre-determined time points or upon complete hind-limb paralysis. Half of the brain was used for immunohistochemistry and was immediately drop-fixed in 4% paraformaldehyde and remained in fixative for at least 48 hours. The other half was dissected into gross brain regions, including the cortex and brain stem, and either snap-frozen on dry ice and stored at −80°C or processed immediately. Half of spinal cords collected remained in the spinal column and were drop-fixed in 4% paraformaldehyde and half were removed and frozen or processed in the same manner as the brains.

### Immunohistochemistry and mapping of αS pathology

All fixed brains were embedded in paraffin and serially sectioned (6 μm) in the sagittal plane. Four to Six sections taken every 12th section, starting at first section from midline containing hippocampus, was processed using standard immunohistochemical (IHC) techniques for DAB (Covance) detection as previously described^8,9,11^. Sections were counterstained with hematoxylin and imaged with a Leica DM2500 microscope using Leica Application Suite v4.1 imaging software. PhosphoSerine-129 αS (pS129αS) positive inclusions/cell bodies and neurites were mapped at three lateral positions: 0.225, 1.10, and 1.525 mm from the midline using Image J/Fiji (NIH) as previously described ^9^.

### Immunofluorescence staining

Paraffin sections of sagittal brain (BrSt injected) and SpC (IM injected) were processed for immunofluorescent staining as previously described^9,17^. Briefly, the tissues were first deparaffinized followed by 30 minutes of antigen retrieval. 3% hydrogen peroxide was used to quench endogenous peroxidases followed by blocking using 100% background sniper solution for 13 minutes. Sections were then incubated in primary antibodies with 5% sniper diluted in TRIS overnight at 4°C (pS129αS, Wako, 015–25191, 1:500; Iba1, Wako, 019–19741, 1:100; GFAP, Dako Cytomation, Z0334, 1:200) followed by incubation in secondary fluorophore conjugated antibody (Donkey anti-mouse 488, Donkey anti-rabbit 594). Sections were imaged using a Leica Stellaris 8 confocal microscope.

#### Western blot (immunoblot) analysis.

Lysate preparation and immunoblot analysis were performed as previously described^9,11^. Briefly, brain tissue from mice were dissected and stored at −80°C and frozen tissues were homogenized in TNE buffer [Tris-HCl 50 mM, NaCl 150 mM and EDTA 5 mM, and protease and phosphatase inhibitors (HALT; Thermo-Fisher; Waltham, MA)]. To obtain total lysate, homogenates were solubilized with 0.5% NP40, 0.5% DOC, and 1% SDS. For TX-100 non-ionic detergent fractionation, TNE homogenates were added to 1% TX-100 and sonicated prior to centrifugation at 100,000×g for 20 min. Supernatant was collected as the soluble fraction while the pellet was washed and centrifuged again in 1% TX-100. The resulting pellet was then reconstituted in TNE with detergents as the TX-100 insoluble fraction^9,25^.

The protein content of the lysates were determined using the BCA assay (Pierce, Thermo; Rockford, IL) and samples were prepared to equal protein concentrations in reducing, SDS-sample, Laemmli buffer (Boston BioProducts; Ashland, MA). For Western blot analysis, protein lysates were run on Criterion^™^ TGX^™^ gels (BioRad, Hercules, CA) and transferred onto nitrocellulose membranes. Proteins on membranes were detected using appropriate primary antibodies followed by horseradish peroxidase (HRP)-conjugated secondary antibodies (Invitrogen, Carlsbad, CA). Membranes were then developed using chemiluminescent substrates (BioRad and Thermo) and imaged using the ImageQuant LAS 4000 detection system (GE Life Sciences, Piscataway, NJ). Densitometry on Western blot images was analyzed using the ImageQuant TL 8.1 software (GE Life Sciences).

### Antibodies

Primary antibodies used for immunohistochemistry and immunoblots are listed in Supplemental Table S1.

### Statistical analysis

All analyses were done using GraphPad Prism. Values are expressed as mean ± SD/SEM as indicated. Differences between means were analyzed by Student’s *t*-test, one-way ANOVA, or two-way ANOVA. ANOVA analyses were combined with the Tukey post-hoc test for multiple comparisons (Prism; Graph Pad Software). Log-rank (Mantel-Cox) test was used to assess significant differences in survival curves. An α value of 0.05 and adjusted *P* values for multiple comparisons were used to determine significance.

## Data Availability

The datasets and images used and/or analyzed for the current study are available from the corresponding author on reasonable request.
